# The effect of climate change on Arcto‐Tertiary Mexican beech forests: Exploring their past, present, and future distribution

**DOI:** 10.1002/ece3.9228

**Published:** 2022-08-22

**Authors:** Fressia N. Ames‐Martínez, Isolda Luna‐Vega, Gregg Dieringer, Ernesto C. Rodríguez‐Ramírez

**Affiliations:** ^1^ Laboratorio de Biotecnología y Biología Molecular Universidad Continental, Urbanización San Antonio Huancayo Peru; ^2^ Laboratorio de Biogeografía y Sistemática Departamento de Biología Evolutiva, Facultad de Ciencias Universidad Nacional Autónoma de México Ciudad de México Mexico; ^3^ Department of Natural Sciences Northwest Missouri State University Maryville Missouri USA; ^4^ Laboratorio de Dendrocronología Universidad Continental, Urbanización San Antonio Huancayo Peru

**Keywords:** ecological refugia, *Fagus mexicana*, palaeoclimatic, species distribution model, suitability habitat

## Abstract

*Fagus mexicana* Martínez (Mexican beech) is an endangered Arcto‐Tertiary Geoflora tree species that inhabit isolated and fragmented tropical montane cloud forests in eastern Mexico. Exploring past, present, and future climate change effects on the distribution of Mexican beech involves the study of spatial ecology and temporal patterns to develop conservation plans. These are key to understanding the niche conservatism of other forest communities with similar environmental requirements. For this study, we used species distribution models by combining occurrence records, to assess the distribution patterns and changes of the past (Last Glacial Maximum), present (1981–2010), and future (2040–2070) periods under two climate scenarios (SSP 3‐7.0 & SSP 5‐8.5). Next, we determined the habitat suitability and priority conservation areas of Mexican beech as associated with topography, land cover use, distance to the nearest town, and environmental variables. By considering the distribution of Mexican beech during different periods and under different climate scenarios, our study estimated that high‐impact areas of Mexican beech forests were restricted to specific areas of the Sierra Madre Oriental that constitute refugia from the Last Glacial Maximum. Regrettably, our results exhibited that Mexican beech distribution has decreased 71.3% since the Last Glacial Maximum and this trend will for the next 50 years, migrating to specific refugia at higher altitudes. This suggests that the states of Hidalgo, Veracruz, and Puebla will preserve the habitat suitability features as ecological refugia, related to high moisture and north‐facing slopes. For isolated and difficult‐to‐access areas, the proposed methods are powerful tools for relict‐tree species, which deserve further conservation.

## Graphical abstract

Suitability habitat Climatic effect on Mexican beech forests

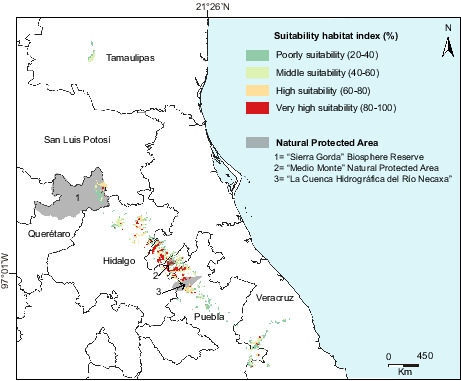

## 1 INTRODUCTION

**FIGURE 1:**
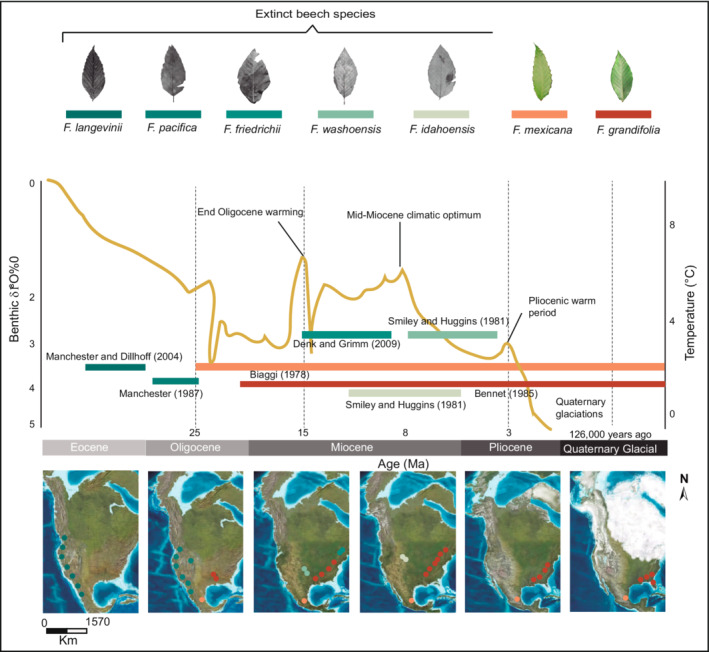
Paleo‐distribution map of North American beech species. Light blue curve shows global average *Δ* ^18^O derived from benthic foraminifera, which mirrors the major global temperature trends from Eocene to Quaternary Glacial (modified from Jiang et al., 2020)

The abrupt ice cover variation that occurred during Eocene–Oligocene (~33.7 Ma BP; Helmer et al., 2019) influenced the global climate by interrupting the cooling trend. During these periods, vast amounts of CO_2_ were emitted into the atmosphere, and evaporation from the sea increased as reflected in benthic foraminifera values (from 1 to 3 oxygen isotope composition [δ^18^O^0/00^]; ~300,000 years; Graham, 1976; Figure 1), influencing hydrological cycles (Tang et al., 2017). During this period, we might have expected an increase in cloudiness although extensive studies have shown a decreasing trend of minor cloud immersion affecting the tropical montane cloud forests (TMCFs) and thus triggering local extinctions via enhanced dryness (Ponce‐Reyes et al., 2012). Identifying the extent of the relict‐endangered plants' response to climate change helps design flexible conservation strategies for Mexican TMCFs. One of the most interesting TMCFs characteristics is their specific floristic diversity (with ~22,800 vascular plant species) and high endemism (Silveira et al., 2019).

Arcto‐Tertiary Geoflora species (sensu Baskin & Baskin, 2016; Chaney, 1959) as currently distributed in the Mexican TMCFs include temperate tree genera with broad ecological tolerances (e.g., *Carya*, *Fagus*, *Liquidambar*, *Liriodendron*, *Magnolia*, *Meliosma*, and *Tilia*; Graham, 1976). Therefore, the Mexican TMCFs have had a stable long‐term climate and remain in critical hotspots (known as Pleistocene glacial refugia; Rico et al., 2021) and possessed a high conservation priority for the long‐term persistence of relict‐forest communities (Tang et al., 2017). Before and during the Ice Ages (~100,000 year‐interval), several temperate deciduous broad‐leaved tree species (e.g., *Quercus tardifolia* C.H. Mull. And *Franklinia alatamaha* Marshall [EW]) and conifer species (e.g., *Picea critchfieldii* Jackson & Weng) were absent from the Northern Hemisphere (Jackson & Weng, 1999; Knapp et al., 2020).

In the Oligo‐Miocene (c. 25 Ma BP), temperate tree genera such as *Fagus* appeared in the Mexican TMCF floristic composition (Graham, 1976). The extension of drought environments into North America influenced further contact among *Fagus* populations in Mexico and the United States (Fang & Lechowicz, 2006; Figure 1). Several geographically isolated forests have shared and exchanged floristic structure and composition during the early Tertiary (~66–2.6 Ma BP). This is the starting point for the current similarity of the American beech between the United States and Canada (Rodríguez‐Ramírez et al., 2021). Nevertheless, the earliest palynological evidence for *Fagus mexicana* Martínez (Mexican beech) in eastern Mexico dates to c. 25 Ma BP (Oligo‐Miocene) from the states of Veracruz and Chiapas (Biaggi, 1978; Graham, 1976, 1999; Palacios Chavez & Rzedowski, 1993). This is where the origin of “modern” terrestrial ecosystems has been documented, appearing in several of the world's hotspots of terrestrial biodiversity (Rahbek et al., 2019).

**FIGURE 2:**
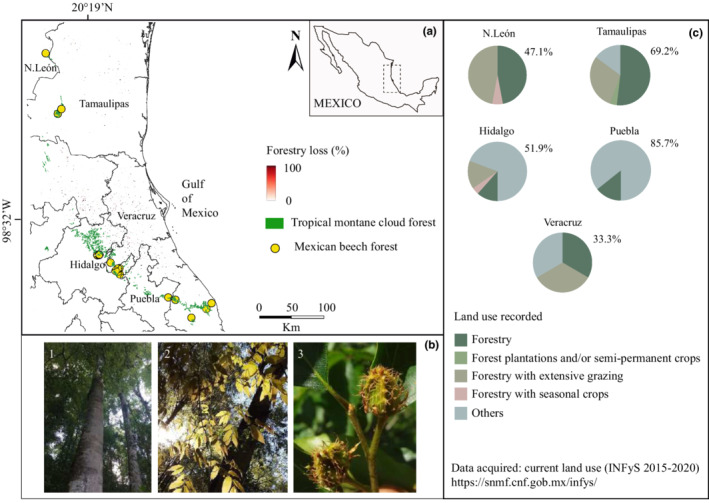
(a) Current distribution of the Mexican beech forest throughout the Sierra Madre Oriental in eastern Mexico. (b) Mexican beech features (1 = tree; 2 = leaves; and 3 = beechnut); and (c) land use recorded on forestry areas

Scattered and small isolated Mexican beech forests (1.647 km^2^; Figure 2; Rodríguez‐Ramírez, Martínez‐Falcón, & Luna‐Vega, 2018) are considered relict‐endemics occurring in eastern Mexican TMCFs. Mexican beech is an unprotected species, although on the IUCN Red List it is cataloged as LC (“Least Concern,” https://www.iucnredlist.org/species/62004694/62004696#population). The Mexican beech forests occur at altitudes of 1509–2034 m above sea level (asl) on northern steep‐ravines with high moisture, on vitric slopes, and near streams harboring unique floristic assemblages with a specific microclimate (Rodríguez‐Ramírez et al., 2018). The Mexican beech is dominant and co‐exists with other Arcto‐Tertiary Geoflora species such as *Magnolia schiedeana* Schltdl., *Clethra mexicana* DC., *Liquidambar styraciflua* L. and several Neotropical oak species (e.g., *Quercus delgadoana* S. Valencia, Nixon & L.M. Kelly, *Q*. *meavei* S. Valencia, Sabas & O.J. Soto and *Q. trinitatis* Trel.), but rarely *Acer saccharum* Marshall and certain conifer species such as *Pinus patula* Schltdl. & Cham., *Podocarpus reichei* J. Buchholz & N.E. Gray, *Picea martinezii* T. F. Patterson and *Taxus globosa* Schldl. Additionally, tree ferns such as *Cyathea fulva* (M. Martens & Galeotti) Fée and *Dicksonia sellowiana* var. *arachneosa* Sodiro (Rodríguez‐Ramírez, Sánchez‐González, & Ángeles‐Pérez, 2018) are moreover common.

In response to global climate change, conservation strategies and decisions regarding the location of protected hotspot areas must consider regional climate changes and their effects on Arcto‐Tertiary Geoflora species range distributions. Unfortunately, there are no suitable studies to assess the potential impact of climate change on Mexican beech forests (Téllez‐Valdés et al., 2006) or conservation strategies considering spatial ecology, temporal patterns, and environmental requirements. Climatic scenarios predict that masting tree species such as the Mexican beech will be affected by climate change, influencing phenological processes at the population level (Rodríguez‐Ramírez et al., 2021). Modeling of species distribution links species presence data with ecological and climate variables, assuming that the known distribution reflects the species' survival pattern (Chardon et al., 2020; Di Pasquale et al., 2020; Hirzel et al., 2001). According to Ponce‐Reyes et al. (2013), Mexican TMCFs distribution will be drastically reduced by over 90% by the year 2080.

We tested the hypothesis that the mountains of eastern‐central Mexico have been sufficiently stable climatically to be considered a long‐term Arcto‐Tertiary Geoflora refuge for many taxa such as Mexican beech. In addition, we theorized that there will be a decline in the extent of suitable habitats for relict‐endemic tree species under future climate change. In this study, our aims were to (1) describe the *past* paleoclimate history of the Mexican beech forests considering specific environmental variables and elevation data; (2) model the *present* Mexican beech potential distribution range as a function of particular environmental variables; (3) explore the *future* Mexican beech forests' potential distribution under climate change scenarios; and (4) determine Mexican beech suitability habitat models and priority areas (hotspots), environmental ranges, and anthropic effects. The exploration of the past, present, and future ecological refugia has the potential to explain their importance for conservation.

## 2 MATERIAL AND METHODS

### 2.1 Study area

To determine the current natural distribution of Mexican beech, we conducted field studies of TMCFs from eastern Mexico over 14 years, from 2007 to 2021. We complemented these studies with satellite imagery observations (Rodríguez‐Ramírez et al., 2021) and occurrence data derived from previous studies (Ehnis, 1981; Montiel‐Oscura, 2011; Rodríguez‐Ramírez et al., 2018, 2021; Williams‐Linera et al., 2000). We selected 15 Mexican beech forests in five states (Figure 2) and determined forest areas and environmental conditions for each state (Table 1). The occurrence data for each selected stand were used to build the past, present, and future prediction models.

**TABLE 1:** Current Mexican beech coverage area from each Mexican state, climatic features, and altitude

State	Current area (km^2^)	Mean precipitation (mm)	Mean temperature (°C)	Altitude (m asl)
Nuevo León	0.340	761.00	24.40	1950–2000
Tamaulipas	0.134	715.50	24.35	55–135
Hidalgo	1.065	1455.56	15.36	2000–2200
Veracruz	0.084	1501.20	17.80	280–2550
Puebla	0.023	1618.00	12.30	1610–1660
Average	1.647	1210.25	18.84	55–2550

### 2.2 Climatic variables

#### 2.2.1 Past climate

To evaluate the paleoclimatic data for the Last Glacial Maximum (LGM, ~21,000 years), we used Paleoclimate Modelling Intercomparison Project 3 (PMIP3), supported by WCRP/CLIVAR/Working Group on Coupled Modelling (WGCM) and IGBP/PAGES (Taylor et al., 2012). We used 19 bioclimatic variables from seven paleoclimatic model projections (e.g., CNRM‐CM5, IPSL‐CM5A‐LR, FGOALS‐g2, MIROC‐ESM, MPI‐ESM‐P, MPI‐CGCM3 y CCSM4) and elevation data. We obtained bioclimatic and elevation data from CHELSA v. 2.0 (http://chelsa‐climate.org/; Karger et al., 2022).

#### 2.2.2 Present climate

We used elevation data and 19 bioclimatic variables from the CHELSA database, which involved a recent time frame (1981–2010), the aridity index, and the annual evapotranspiration variables from the CGIAR‐CSI website (www.cgiar‐csi.org; Trabucco & Zomer, 2019), with a resolution layer of c. 1 km^2^. We selected bioclimatic variables for the Mexican beech based on predictive maps following two different approaches, one statistically based (Fielding & Bell, 1997; Peterson & Soberón, 2012) and the other using Mexican beech occurrence records (Ehnis, 1981; Pérez‐Rodríguez, 1999; Rodríguez‐Ramírez et al., 2013, 2021; Rowden et al., 2004).

#### 2.2.3 Future climate

To assess the climate projection for the time frame 2040–2070, we used the Coupled Model Intercomparison Project phase 6 (CMIP6) of the WGCM (Eyring et al., 2016). We used 19 bioclimatic variables for the five models to derive future climate projections (GFDL‐ESM4, IPSL‐CM6A‐LR, MPI‐ESM1‐2‐HR, MRI‐ESM2‐0, and UKESM1‐0‐LL). Lastly, we obtained climate data from the CHELSA database (Karger et al., 2022).

We selected two future climate scenarios (Shared Socioeconomic Pathways; SSP 3‐7.0 and 5‐8.5) under two greenhouse gas concentration regimes and without implementing future climate policies (e.g., Kyoto Protocol; Bosso et al., 2017; Xian et al., 2022). The SSP 5‐8.5 scenario assumes high greenhouse gas concentrations throughout the 21st century, reaching equilibrium by 2100, whereas the SSP 3‐7.0 scenario represents global greenhouse gas concentrations peaking in 2070. We assumed that SSP 5‐8.5 would be the most chaotic scenario if we assume no measures are taken to avoid the climate effects; SSP 3‐7.0 would be a less chaotic scenario, assuming emissions reductions occur. Finally, to assess the climate change effects on the Mexican beech distribution in TMCF of eastern Mexico, we developed an species distribution models (SDM) as a function of the environmental variables considered above in the five selected models, for the SSP 3‐7.0 and SSP 5‐8.5 scenarios.

### 2.3 Species distribution modeling

We estimated the relationship between environmental variables and Mexican beech presence using Pearson correlations (*r* < .70), retaining those relationships. Additionally, we performed a Jackknife analysis that incorporated the following options: False discovery rate calculation, the multicollinearity degree, coefficient of determination of linear regression, tolerance, variance inflation factor, the Bayesian information criterion (BIC), and Akaike (AIC). Finally, we combined this with our knowledge of Mexican beech responses to specific environmental factors (i.e., mean annual temperature, mean temperature of the warmest quarter, mean temperature of the coldest quarter, annual precipitation, and altitude; Fang & Lechowicz, 2006; Peters, 1997). We performed the analysis using the software R v. 4.1.2 with *fuzzySim* and *sdm* packages (Barbosa, 2021; Naimi & Araújo, 2016).

Assessment of the best candidate model was performed using the *KUENM* package (Cobos et al., 2019). We derived the potential distribution from the best model, using the average performance evaluation indicators (AUC), partial ROC (receiver operating characteristic), omission rate, and the optimal complexity parameter (AIC‐Akaike Information Criterion; Bozdogan, 1987; Elith & Leathwick, 2009; Gutiérrez et al., 2018). We followed a logistic threshold for training presence clipping which corresponds to the 10% of data with the lowest probability value which is commonly used in conservation studies (Abba et al., 2012; Ancillotto et al., 2019; Khanghah et al., 2022). In addition, we used the trimming threshold (~24%–26%) of the present model, which approximates the distribution of the species according to Williams‐Linera et al. (2000) and Rodríguez‐Ramírez et al. (2021). We then selected the models from the “best” variable set (500), scenario, and candidate model (Appendix S1).

We determined the surface in each climatic model (km^2^), assessing the surface variation among *past*, *present*, and *future* scenarios (Appendix S2). Next, we overlayed the Mexican beech geographic models and then divided the probabilities of occurrence into five categories: (1) absent, 0% to threshold values; (2) low, threshold values to 40%; (3) intermediate, 40%–60%; (4) high, 60%–80%; and (5) very high, 80%–100%. Lastly, we calculated the final shapes using the software MaxEnt v.3.4.4 (Philips et al., 2004), with maps edited in QGIS v.3.18.3 (QGIS.org, 2021).

We followed the ODMAP protocol (Overview, Data, Model, Assessment and Prediction; Appendix S3; Zurell et al., 2020) of the modeling process, as its components reflect the main steps for SDM, assessing the model quality and allowing peer review.

### 2.4 Predicted Mexican beech suitability habitat

We linked *present* and *future* potential Mexican beech distribution with suitable habitats, recognizing appropriate grids in the SSP 3‐7.0 and SSP 5‐8.5 scenarios. In addition, we used specific environmental variables as coverage shapefiles (Global Forest Change, https://earthenginepartners.appspot.com/; Hansen et al., 2013), land cover use (CONABIO, http://www.conabio.gob.mx/informacion/metadata/gis/nalcmsmx05gw.xml?_httpcache=yes&_xsl=/db/metadata/xsl/fgdc_html.xsl&_indent=no; Ocaña, 2010), and distance to the nearest town (https://datos.gob.mx/busca/dataset?theme=Geoespacial; Registro Agrario Nacional, 2019).

We performed a spatial distribution bias correction to avoid over‐adjusting future projections in the SDM. We included 10,000 bias files (points where the species is not recorded) and environmental variables to assess potential habitat suitability analysis. We achieved the analysis with the SDMtoolbox package in ArcGIS v. 10.8 (Brown et al., 2017). We implemented a Gaussian Kernel (Bosso et al., 2022; Mushtaq et al., 2021; Zhang et al., 2018) using QGIS software to avoid a sampling bias and help identify the highest potential suitability areas. With this, we selected the high suitable priority areas (hotspots) for conservation.

## 3 RESULTS

### 3.1 Mexican beech potential distribution under paleoclimate (LGM)

Under IPSL‐CM5A‐LR projection for the LGM, we demonstrated that Mexican beech was distributed through the Sierra Madre Oriental of eastern Mexico with a coverage of 7388.51 km^2^ and detected that Mexican beech previously covered 249.07% of the current distribution (~2116.65 km^2^). The environmental variables which influenced the presence of the species were altitude (31.5%), mean temperature of warmest quarter (BIO10, 17.9%), annual precipitation (BIO12, 17.5%), and precipitation of wettest quarter (BIO16, 10.9%), accounting for almost 77.8% of the explained variation (Appendix S3A). In addition, we detected that Mexican beech was paleoclimatically distributed in the states of Nuevo León (10.97 km^2^), Tamaulipas (1550.71 km^2^), San Luis Potosí (351.43 km^2^), Querétaro (177.06 km^2^), Hidalgo (1039.16 km^2^), Veracruz (2397.81 km^2^), and Puebla (1861.37 km^2^; Table 2).

**TABLE 2:** Potential area coverage of the climate model (*present*, *past*, and *future*) in Mexican beech distribution

State	Present (km^2^)	Past (km^2^)	Loss past (%)	SSP 3‐7.0 (km^2^)	Gain or loss SSP 3‐7.0 (%)	SSP 5‐8.5 (km^2^)	Gain or loss SSP 5‐8.5 (%)
Coahuila de Zaragoza	–	–	–	38.74	+3874.00	1.55	155
Nuevo León	0.78	10.97	−0.48	124.87	+15,909	123.62	15,748
Tamaulipas	44.14	1550.71	−71.18	12.54	−72.00	53.50	21.21
San Luis Potosi	49.40	351.43	−14.27	29.5	−40.00	5.58	−88.70
Querétaro	79.74	177.06	−4.60	64.60	−19.00	31.09	−61.00
Hidalgo	727.13	1039.16	−14.74	192.57	−74.00	30.51	−95.80
Veracruz	696.76	2397.81	−80.37	595.26	−15.00	891.95	28.01
Puebla	518.70	1861.37	−63.43	650.15	+25.00	172.69	−66.71
Oaxaca	–	–	–	45.72	+4572.00	0.00	0
Total	2116.65	7388.51	−249.07	1753.95	−17.13	1310.49	−38.09

### 3.2 Current distribution, model performance, and Mexican beech potential distribution under the present climate

According to our *present* model, the potential range for Mexican beech was about ~2116.65 km^2^. Notably, areas in the TMCFs of eastern Mexico showed suitability for Mexican beech under current records. The detected distribution was as follows: 0.78 km^2^ for Nuevo León, 44.14 km^2^ for Tamaulipas; 49.40 km^2^ for San Luis Potosí, 79.74 km^2^ for Querétaro; 727.13 km^2^ for Hidalgo; 518.70 km^2^ for Puebla; and 696.76 km^2^ for Veracruz (Table 2).

We projected suitable habitats for Mexican beech as expressed by the occurrence probability (AUC = 0.9993 ± 0.0614). The Jackknife analysis detected that the mean annual temperature (BIO1), seasonality of temperature (BIO4), annual precipitation (BIO12), seasonality of precipitation (BIO15), precipitation of the driest quarter (BIO17), precipitation of the coldest quarter (BIO19), aridity index, and evapotranspiration as the most relevant environmental variables determining potential presence (Appendix S4). According to the current potential distribution, we identified that evapotranspiration (26.9%), mean annual temperature (BIO1; 16.8%), and precipitation of the driest quarter (BIO19; 12.7%) were the most relevant factors for the presence of Mexican beech (~56.4% of the model prediction). We suggest the potential presence of the species in five Natural Protected Areas, such as the “El Cielo” Biosphere Reserve, Tamaulipas; “Sierra Gorda” Biosphere Reserve, Querétaro; Metztitlán Canyon Biosphere Reserve, Hidalgo; Cuenca Hidrográfica del Río Necaxa Protected Forest Zone, Puebla; and Cofre de Perote National Park, Veracruz. In addition, some unprotected natural areas in Hidalgo, Puebla, and Veracruz support the model (Appendix S5).

### 3.3 Mexican beech potential distribution under different future climate scenarios (2040–2070)

We restricted the potential predictions of suitable environments to hotspot areas within the current Mexican beech forest distribution, assuming that it cannot migrate to new TMFC fragments in the short time span of this analysis (50 years). Climate change effects on future Mexican beech forest distribution from 2040 to 2070 were estimated under SSP 3‐7.0 and 5‐8.5 scenarios with both scenarios showing a significant decrease in suitable habitat. Mexican beech detected a loss of habitat suitability: 17.13% (1753.95 km^2^) under the SSP 3‐7.0 of the IPSL‐cm6a‐lr model and 38.06% (1310.49 km^2^) under SSP 5‐8.5 of the MRI‐esm2‐0 model (Appendix S2 and S6). This loss occurred in at least some TMCFs projected to be suitable for Mexican beech forests.

**FIGURE 3:**
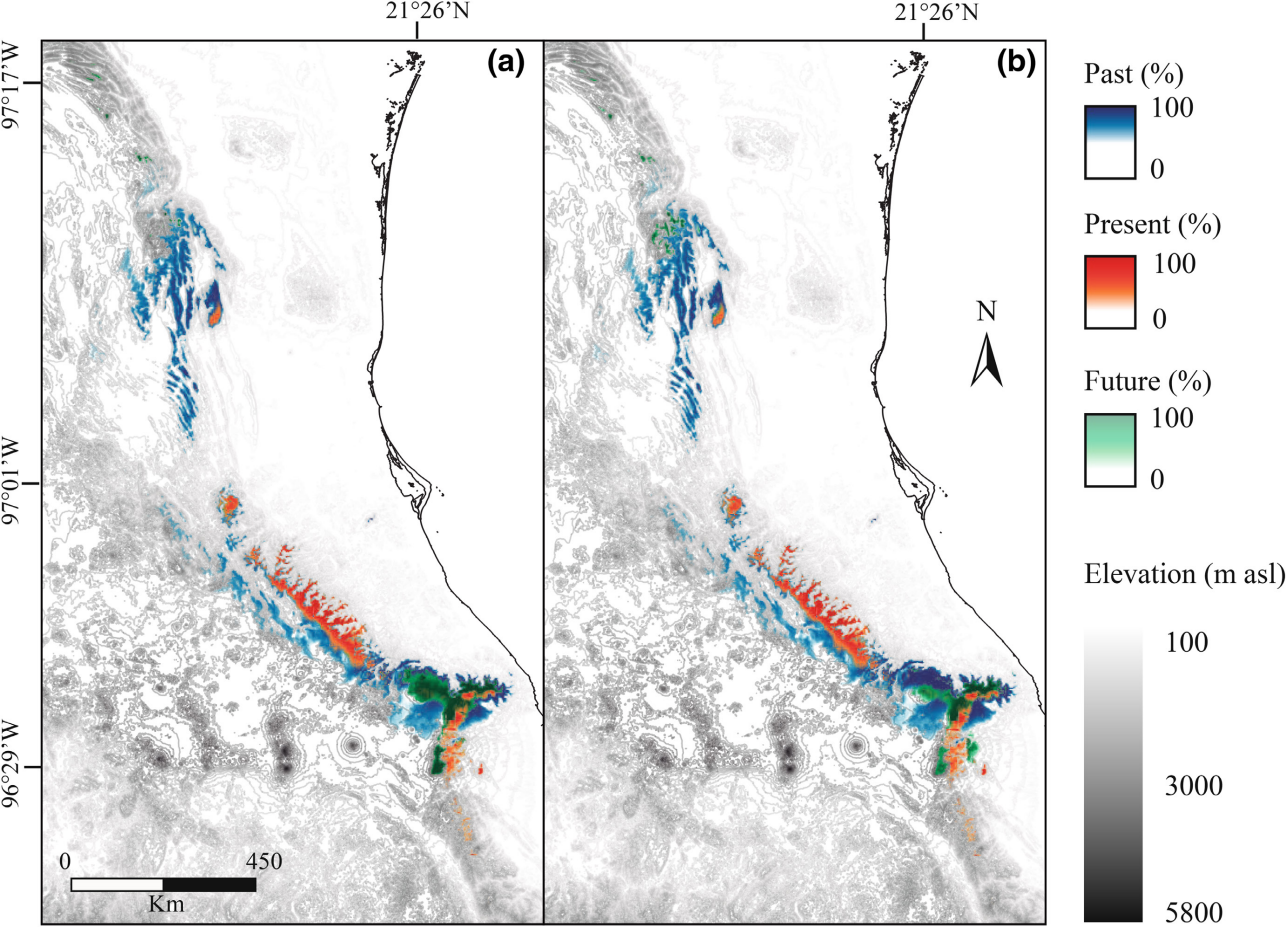
Overlapping habitat of the Mexican beech from *past*, *present*, and *future* climate projections. (a) SSP3‐7.0; and (b) SSP5‐8.5 climate scenarios

Environmental variables that contributed to the SSP 3‐7.0 scenario for the *future* (>80.4%) were the maximum temperature of the warmest month (BIO5; 44.1%), mean diurnal range (BIO2; 14.1%), precipitation of driest month (BIO14, 13.3%), and precipitation seasonality (BIO15, 8.9%). Under the SSP 5‐8.5 scenario, the more representative environmental variables were (>84.8%) as follows: maximum temperature of the warmest month (BIO5; 45.0%), precipitation of the driest month (BIO14; 18.6%), precipitation of the wettest month (BIO13; 11.4%), and mean diurnal range (BIO2, 9.8%; Appendix S4B). In addition, we detected an altitudinal increase in the current distribution from 1300 to 2200 m asl. We demonstrated an increment of altitudinal range about the projected *future* climate scenarios from 1700 to 3025 m asl for the SSP 3.7‐0 scenario (Figure 3a) and from 1400 to 2850 m asl for the SSP 5.8‐5 scenario (Figure 3b).

Under SSP 3‐7.0 and SSP 5‐8.5 scenarios, we detected suitable habitats in nine Mexican states, mainly in Veracruz (595.26–891.95 km^2^), Puebla (650.15–172.69 km^2^) and Hidalgo (192.57–30.51 km^2^; Table 2). Likewise, we identify two new potential suitable habitats; (1) the state of Coahuila (38.74–1.55 km^2^; ~25°12′52.28″N, 100°14′41.59″W; 1749–1753 m asl); and (2) Sierra de Juárez in the state of Oaxaca (45.72 km^2^; ~17°24′43.32″N, 96°29′48.26″W; 2944–3025 m asl; Table 2), where no current record exists for Mexican beech.

### 3.4 Priority conservation areas

**FIGURE 4:**
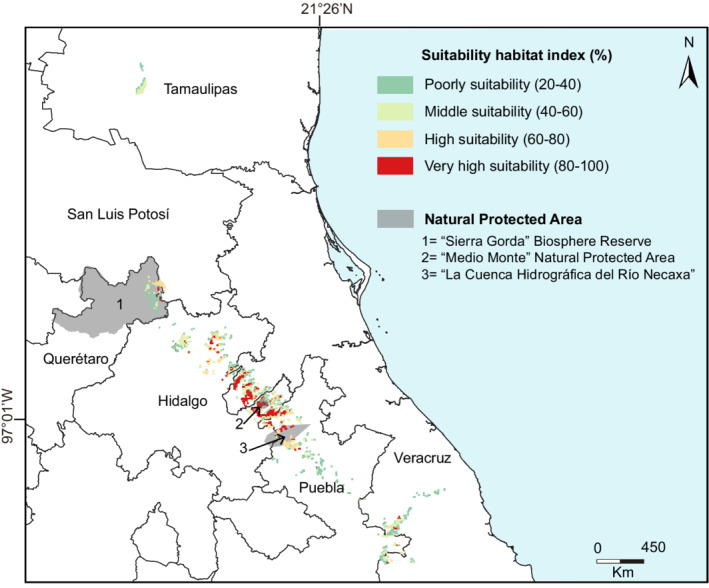
Mexican beech habitat suitability under *present* and *future* climate change scenarios predicted with our approach

The Mexican beech habitat suitability analysis detected (*present* and *future* models) an area of 316.31 km^2^, highlighting the states of Hidalgo (145.5 km^2^), Puebla (55.27 km^2^), Veracruz (111.47 km^2^), Querétaro (3.66 km^2^), and San Luis Potosí (0.41 km^2^). Further, we identified hotspots covering the “Sierra Gorda” Biosphere Reserve (Querétaro; 4.69 km^2^), “Medio Monte” Natural Protected Area (Hidalgo; 1.52 km^2^), and “La Cuenca Hidrográfica del Río Necaxa” Closed Forest Protection Zone (Puebla; 13.42 km^2^; Table 3; Figure 4). We detected hotspots outside of Natural Protected Areas, which represent 90.41% of the potential Mexican beech habitat suitability. Here, we detected specific environmental variables that influence habitat suitability which included: mean annual temperature (BIO1; from 6 to 18°C), annual precipitation (BIO12; from 1000 to 2450 mm), precipitation of coldest quarter (BIO19; from 75 to 190 mm), and evapotranspiration (from 1000 to 1650 mm/day). These results indicate that Mexican beech occurs is comprised of moist environments (Cwb; Peel et al., 2007; Figure 4).

**TABLE 3:** Suitability habitat coverage in each Mexican state and Protected Natural Areas

	Surface (km^2^)
State	
Hidalgo	145.50
Puebla	55.27
Veracruz	111.47
Querétaro	3.66
San Luis Potosí	0.41
Total	316.31
Protected natural areas	
“Sierra Gorda” Biosphere Reserve	4.69
“La Cuenca Hidrográfica del Río Necaxa” Forest Protection Zone Closed	13.42
“Medio Monte” Natural Protected Area	1.52
Total	19.63

## 4 DISCUSSION

Patterns of species distribution reflect the interaction between many factors, such as microenvironmental, elevation, anthropic activities, intra‐ and interspecific relations, topography, and physiological features of the tropical species (Báez et al., 2022; Rahbek et al., 2019). In this comprehensive study, we considered specific environmental variables (Fang & Lechowicz, 2006; Rodríguez‐Ramírez, Sánchez‐González, & Ángeles‐Pérez, 2018) to simulate the potential distribution for Mexican beech (*Fagus mexicana*). Our results showed that during the LGM, Mexican beech remained in possible refugia through projected *past* distributions, influenced mainly by precipitation of the wettest month, precipitation of the coldest month, and altitude. In this situation, the climate became cool temperate (~3°C) and climax forest communities were dominated by subtropical evergreen forests with Arcto‐Tertiary Geoflora genera such as *Liquidambar*, *Acer*, *Meliosma*, *Tilia*, *Magnolia* and *Fagus* having migrated to middle latitudes (from 19 to 23°N) during the Eocene (Baskin & Baskin, 2016; Peters, 1997; Steinthorsdottir et al., 2021; Figure 1). Further palynological studies are essential to determine the possible presence of the Mexican beech in potential historical areas of distribution.

The above hypotheses suggest that the recent Mexican beech distribution existed before the climate slowly cooled toward a series of Ice Ages (Peters, 1997). Our models of *past* distribution exhibited that the species covered an area of 7388.51 km^2^, resulting from the reduced availability of suitable habitats brought about by climatic fluctuations. This interpretation is supported by palynological records (Biaggi, 1978; Graham, 1999; Palacios Chavez & Rzedowski, 1993). At present, we have detected an archipelagic distribution that maintains specific Mexican beech ecological refugia (e.g., Hidalgo, Veracruz and Puebla), which is supported by the suitability habitat analysis (Figure 4). According to the current Mexican beech forest coverage (1.647 km^2^, Table 1), our potential distribution models exhibited an area of 2116.65 km^2^, which indicates the possibility of new suitable habitat areas, such as “Ejido La Selva” Conservation Area, Huayacocotla, Veracruz (20°35′N; 98°29′W: Numa Pavón, personal communication) and Pahuatlán, Puebla (20°16′N; 98°09′W; Francisco Vega, personal communication), potentially suitable areas that remain to be explored, or locally where it has become extinct (Quijano et al., 2016) validating the generated model (Figure 4). Therefore, it is possible that ecological niche conservatism has influenced the persistence of the *Fagus* species worldwide (Cai et al., 2021), which limits the distribution of ecologically dissimilar lineages among geographic regions (Jiang et al., 2020; Wiens & Graham, 2005). These regions have complex topographies and specific microclimate factors (e.g., fog, moisture, and mild temperature conditions; 14.8–15.6°C) throughout the Sierra Madre Oriental (Rodríguez‐Ramírez et al., 2021).

Climate change represents a significant potential threat to the future existence of TMCFs (Los et al., 2021). Future projections have revealed Mexican TMCFs would face a dramatic range reduction (68%; Jiménez‐García & Peterson, 2019; Ponce‐Reyes et al., 2012). Projected climatic conditions will cause an increase in temperature (4.1–5°C above current temperature) and CO_2_ (70.04 gigatons for SSP 3‐7.0 and 116.8 gigatons for SSP 5‐8.5; Taylor et al., 2012). Our results confirmed that the Mexican beech forests will likely reduce its range, based on our climate change projections through 2070 (>38% of its potential current extent), by more than 80% over the next 50 years. Mexican beech forests could prefer isolated mountainous regions of the Sierra Madre Oriental with more suitable moisture and temperature conditions (Cai et al., 2021; Fang & Lechowicz, 2006). The above ideas confirm the high climatic sensitivity of Mexican beech to climate change, in particular to drought periods, increasing its extinction risk (Rodríguez‐Ramírez et al., 2018; Téllez‐Valdés et al., 2006).

The *future* climate models developed here have allowed us to identify suitable areas with environmental characteristics for the migration of Mexican beech forests and to propose new conservation areas. Likewise, we detected a high reduction in the presence of Mexican beech, as related to its potential current range, in the states of Hidalgo (>95.8%) and San Luis Potosí (>88.7%), as indicated in the SSP 3‐7.0 and 5‐8.5 scenarios. We developed these scenarios with information on the increase in gases such as carbon dioxide (CO_2_), methane (CH_4_), and nitrous oxide (N_2_O) generating the greenhouse effect and having the effect of increasing temperature (Meinshausen et al., 2020). Nevertheless, current anthropogenic activities (e.g., grazing, illegal logging, and corn/avocado plantations) are expected to directly influence the reduction in this type of ecosystem as well (Quijano et al., 2016; Rodríguez‐Ramírez et al., 2013; Williams‐Linera et al., 2000). Despite these projections, we have detected three Mexican states (Coahuila, Nuevo León, and Oaxaca) with suitable environmental conditions (e.g., altitude, north‐facing slopes, and high moisture) for the existence of the Mexican beech. A similar situation has been described for several beech species worldwide, such as *Fagus orientalis* Lipsky from Turkey (Ayan et al., 2022), *Fagus grandifolia* Ehrh. from the United States (Casajus et al., 2016), *Fagus sylvatica* L. from Spain (Del Río et al., 2018), *Fagus crenata* Blume from Japan (Nakao et al., 2013), and *Fagus longipetiolata* Seemen from China (Yin & Zhou, 2015). These beech forests have not been thoroughly explored and may be considered suitable refugia for the species in future.

By assessing suitable habitat for Mexican beech, we have detected a restricted distribution in Hidalgo, Veracruz, and Puebla as projected by *present* and *future* climatic models. The isolated remnant stands of Mexican beech likely represent refugia, areas characterized by specific microenvironmental factors (e.g., high environmental moisture, steep north‐facing, and temperature), that were once common during Oligo‐Miocene thereby protecting them from climatic change. In analyzing the SDMs, we have found a valuable tool that complements palaeoclimatic (e.g., temperature increase) and paleoecological effects (e.g., palynological records) in understanding historical, current, and future distribution of Mexican beech. This has allowed us to identify suitable new areas with specific environmental conditions that can influence Mexican beech forest conservation.

### 4.1 Management and conservational implications

The Mexican beech presents preservation conflicts because of anthropic activities such as beechnut harvesting, grazing, illegal logging, and corn/avocado plantations (Rodríguez‐Ramírez et al., 2013; Williams‐Linera, 2007). Our results exhibit that Mexican beech distributions projected from ecological niche models (*past*, *present*, and *future*) can provide a realistic potential geographic range proxy, and habitat suitability to identify ecological refugia. Nevertheless, it is necessary to consider connectivity among fragments as reported by Rodríguez‐Ramírez et al. (2013) for Hidalgo Mexican beech forests, and population disequilibrium as shown in other Mexican TMCF tree species (e.g., *Liquidambar styraciflua* L., Ruiz‐Sanchez & Ornelas, 2014; *Podocarpus* spp., Ornelas et al., 2019; *Magnolia schiedeana*, Rico et al., 2021) from the perspective of autoecology, which is essential for management and conservation implications, with emphasis on the ecological refugia as was recorded in this study.

## AUTHOR CONTRIBUTIONS

**Fressia N. Ames‐Martínez:** Conceptualization (lead); data curation (lead); formal analysis (lead); investigation (equal); methodology (lead); writing – original draft (equal); writing – review and editing (equal). **Isolda Luna‐Vega:** Funding acquisition (lead); project administration (lead); writing – review and editing (equal). **Gregg Dieringer:** Writing – original draft (equal); writing – review and editing (equal). **Ernesto C. Rodríguez‐Ramírez:** Conceptualization (supporting); formal analysis (supporting); investigation (lead); writing – original draft (lead); writing – review and editing (equal).

## CONFLICT OF INTEREST

The authors declare no conflict of interest.

## Supporting information


Appendix S1‐S6

## Data Availability

Scripts are available at 10.6084/m9.figshare.20233758 to replicate the analyses of this study. Furthermore, the species occurrence points are available in Rodríguez‐Ramírez et al. (2021) and at the Global Biodiversity Information Facility online database (GBIF; https://www.gbif.org/). The climatic variables can be found on the CHELSA database (http://chelsa‐climate.org/).
