# Supplementary material for: The effect of climate change on Arcto‐Tertiary Mexican beech forests: Exploring their past, present, and future distribution
Source: Ecol Evol. 2022 Aug 22;12(8):e9228. doi: 10.1002/ece3.9228 (PMC9395944; doi:10.1002/ece3.9228)
Supplement: Supplementary file 1 — Appendix S1‐S6 [file ECE3-12-e9228-s001.docx]

**Ecology and Evolution**

**SUPPORTING INFORMATION**

**The effect of climate change on Arcto-Tertiary Mexican beech forests: exploring their past, present, and future distribution**

Fressia N. Ames-Martínez, Isolda Luna-Vega, Gregg Dieringer and Ernesto C. Rodríguez-Ramírez

Appendix S1. Mathematical indicators of each chosen best model.

| Model | Parameters | Mean AUC ratio | Partial ROC | Omission rate at 5% | AICc | Delta AICc | W AICc |
| --- | --- | --- | --- | --- | --- | --- | --- |
| CCSM4_1.0_F_lqh_Set4 | Features: Linear, quadratic, hinge.  Regulation multiplier:1.0  Variables set:4 | 1.98254 | 0.000 | 0.000 | 547.21 | 1.245 | 0.0024 |
| CNRM-CM5_1.0_F_lqh_Set18 | Features: Linear, quadratic, hinge.  Regulation multiplier:1.0  Variables set:18 | 1.98246 | 0.000 | 0.000 | 568.12 | 1.325 | 0.0045 |
| FGOALS-g2_1.0_F_lqh_Set44 | Features: Linear, quadratic, hinge.  Regulation multiplier:1.0  Variables set:44 | 1.98237 | 0.000 | 0.000 | 574.82 | 0.547 | 0.0037 |
| IPSL-CM5A-LR_1.0_F_lqh_Set0 | Features: Linear, quadratic, hinge.  Regulation multiplier:1.0  Variables set:0 | 1.98213 | 0.000 | 0.000 | 532.71 | 0.000 | 0.0068 |
| MIROC-ESM_1.0_F_lqh_Set24 | Features: Linear, quadratic, hinge.  Regulation multiplier:1.0  Variables set:24 | 1.98269 | 0.000 | 0.000 | 546.23 | 0.751 | 0.0036 |
| MPI-ESM-P_1.0_F_lqh_Set3 | Features: Linear, quadratic, hinge.  Regulation multiplier:1.0  Variables set:3 | 1.98254 | 0.000 | 0.000 | 541.27 | 1.265 | 0.0044 |
| MRI-CGCM3_1.0_F_lqh_Set46 | Features: Linear, quadratic, hinge.  Regulation multiplier:1.0  Variables set:46 | 1.98238 | 0.000 | 0.000 | 554.23 | 1.234 | 0.0032 |
| ESM4_1.5_F_lqh_SSP370_Set32 | Features: Linear, quadratic, hinge.  Regulation multiplier:1.5  Variables set:32 | 1.98272 | 0.000 | 0.000 | 546.54 | 1.564 | 0.0045 |
| IPSL-cm6a-lr_1_F_lqh_SSP370_Set25 | Features: Linear, quadratic, hinge.  Regulation multiplier:1  Variables set:25 | 1.98217 | 0.000 | 0.000 | 532.74 | 0.000 | 0.0057 |
| MPI-ESM-1-2-hr_1_F_lqh_SSP370_Set19 | Features: Linear, quadratic, hinge.  Regulation multiplier:1  Variables set:19 | 1.98308 | 0.000 | 0.000 | 539.72 | 0.084 | 0.0044 |
| MRI-esm-2-0_0.5_F_lqh_SSP370_Set41 | Features: Linear, quadratic, hinge.  Regulation multiplier:0.5  Variables set:41 | 1.98254 | 0.000 | 0.000 | 547.21 | 1.243 | 0.0033 |
| UKESM1-0-ll_1_F_lqh_SSP370_Set37 | Features: Linear, quadratic, hinge.  Regulation multiplier:1  Variables set:37 | 1.98235 | 0.000 | 0.000 | 541.57 | 1.347 | 0.0041 |
| ESM4_1_F_lqh_SSP585_Set12 | Features: Linear, quadratic, hinge.  Regulation multiplier:1  Variables set:12 | 1.98227 | 0.000 | 0.000 | 536.17 | 1.597 | 0.0038 |
| IPSL-cm6a-lr_1_F_lqh_SSP585_Set42 | Features: Linear, quadratic, hinge.  Regulation multiplier:1  Variables set:42 | 1.98209 | 0.000 | 0.000 | 534.85 | 1.475 | 0.0048 |
| MPI-ESM-1-2-hr_2_F_lqh_SSP585_Set27 | Features: Linear, quadratic, hinge.  Regulation multiplier:2  Variables set:27 | 1.98267 | 0.000 | 0.000 | 537.49 | 0.015 | 0.0056 |
| MRI-esm-2-0_1_F_lqh_SSP585_Set22 | Features: Linear, quadratic, hinge.  Regulation multiplier:1  Variables set:49 | 1.98247 | 0.000 | 0.000 | 531.28 | 0.000 | 0.0089 |
| UKESM1-0-ll_1_F_lqh_SSP585_Set46 | Features: Linear, quadratic, hinge.  Regulation multiplier:1  Variables set:46 | 1.98226 | 0.000 | 0.000 | 543.97 | 1.489 | 0.0035 |

Appendix S2. Predicted potential distribution of Mexican beech and general performance of the present, past, and future models. Negative values indicate retraction of Mexican beech forests according to current distribution.

| **Period** | **Model** | **SSP** | **AUC train** | **AUC test** | **Surface (km^2^)** | **Difference with present** | |
| --- | --- | --- | --- | --- | --- | --- | --- |
|  |  |  |  |  |  | **km^2^** | **(%)** |
| Present | - | - | 0.9993 | 0.9276 | 2116.65 | - | - |
| Past | CCSM4 | - | 0.9992 | 0.9726 | 43204.99 | 41088.34 | 1941.20 |
|  | CNRM-CM5 | - | 0.9992 | 0.9823 | 17114.36 | 14997.71 | 708.56 |
|  | FGOALS-g2 | - | 0.9992 | 0.9389 | 9297.46 | 7180.81 | 339.25 |
|  | IPSL-CM5A-LR | - | 0.9992 | 0.9860 | 21980.14 | 19863.49 | 938.44 |
|  | MIROC-ESM | - | 0.9992 | 0.8243 | 24970.65 | 22854.00 | 1079.73 |
|  | MPI-ESM-P | - | 0.9987 | 0.9980 | 22253.74 | 20137.09 | 951.37 |
|  | MRI-CGCM3 | - | 0.9987 | 0.9919 | 13158.53 | 11041.88 | 521.67 |
|  | Average | - | 0.9991 | 0.9563 | 21711.41 | 19594.76 | 925.74 |
| Future | ESM4 | 3-7.0 | 0.9972 | 0.9572 | 16951.97 | 13580.10 | 641.82 |
|  | IPSL-cm6a-lr | 3-7.0 | 0.9987 | 0.9935 | 1753.95 | -362.42 | -17.13 |
|  | MPI-esm-1-2-hr | 3-7.0 | 0.9983 | 0.9154 | 14637.17 | 12521.30 | 591.78 |
|  | MRI-esm2-0 | 3-7.0 | 0.9928 | 0.8739 | 59747.59 | 57631.72 | 2723.78 |
|  | UKESM1-0-ll | 3-7.0 | 0.9980 | 0.9972 | 27863.88 | 25748.01 | 1216.90 |
|  | Average | 3-7.0 | 0.9970 | 0.9470 | 24801.44 | 22685.57 | 1072.16 |
|  | ESM4 | 5-8.5 | 0.9950 | 0.8370 | 21870.16 | 19754.29 | 933.62 |
|  | IPSL-cm6a-lr | 5-8.5 | 0.9992 | 0.9918 | 12967.17 | 10851.30 | 512.85 |
|  | MPI-esm-1-2-hr | 5-8.5 | 0.9940 | 0.8332 | 25327.33 | 23211.46 | 1097.02 |
|  | MRI-esm2-0 | 5-8.5 | 0.9790 | 0.8750 | 1310.495 | -805.375 | -38.06 |
|  | UKESM1-0-ll | 5-8.5 | 0.9971 | 0.9018 | 14049.28 | 11933.41 | 564.00 |
|  | Average | 5-8.5 | 0.9930 | 0.8877 | 16699.23 | 14583.36 | 689.24 |

Appendix S3: ODMAP protocol for reporting Mexican beech distribution models following Zurell et al. (2020).

| **ODMAP elements** | **Contents** |
| --- | --- |
| **OVERVIEW** | |
| Authorship | - Authors: Fressia Nathalie Ames Martínez, Isolda Luna Vega, Gregg Dieringer and Ernesto Chanes Rodríguez Ramírez. - Contact email: [echanes@ciencias.unam.mx](mailto:echanes@ciencias.unam.mx).   Title: The effect of climate change on Arcto-Tertiary Mexican beech forests: exploring their past, present, and future distribution. |
| Model objective | Model objective: Mapping and interpolation.  Target output: Suitability habitat index (probability of species presence). |
| Taxon | *Fagus mexicana*, plants. |
| Location | Sierra Madre Oriental, Mexican tropical montane cloud forests. |
| Scale of analysis | Spatial extent: -96.802578, -100.042749, 19.326850, 24.732921 (xmin, xmax, ymin, ymax), covering ~660 km.  Spatial resolution: ~1 km x 1 km.  Temporal extent: past (Last Glacial Maximum), present (1980 – 2019) and future (2040-2070).  Boundary: natural, political. |
| Biodiversity data overview | - Observation type: standardized monitoring. - Response/Data type: presence-only. |
| Type of predictors | Climatic, habitat. |
| Conceptual model/Hypotheses | We hypothesize that the mountains of eastern-central Mexico have been climatically stable to be long-term Arcto-Tertiary Geoflora refugia of many taxa as *Fagus mexicana*. Also, we theorize that there would be a decline in the area extent of suitable habitats for relict-endemic tree species under future climate change. |
| Assumptions | The past, current and future Mexican beech distribution is not at equilibrium with the environment and is mostly driven by climate. There is no sampling bias in the species occurrence data.   - Relevant ecological drivers (or proxies) of species distributions are comprised. - Species are at (pseudo-) equilibrium with their environment. - Species show similar responses to the bait and the sampling instrument. - Sampling is adequate and representative (and any biases are accounted for/corrected). |
| SDM algorithms | - Modelling techniques: MaxEnt. - Model complexity: MaxEnt models were built with linear, quadratic, product, threshold, and hinge features. - Ensembles: We combined model types to form ensemble predictions, we used KUENM package for model selections. |
| Model workflow | We got 16 Mexican beech forest occurrence records from the Sierra Madre Oriental, and fitted ENMs using the KUENM package (Cobos et al. 2019) with MaxEnt algorithm and one candidate predictor set (environmental).  Model calibration was done with careful consideration of data limitations, and the effects of various assumptions available were assessed via experimentation.  We combined model predictions into ensembles using a weighted KUENM algorithm. We evaluated predictive performance using AUC, Partial ROC (Receiver Operating Characteristic), omission rate, and the optimal complexity parameter. We then calculated predicted range sizes from the binary maps and range sizes from the environmental-only, and suitability habitat we calculated with climate, vegetation cover, and distance to the nearest town. |
| Software, codes, and data | - Software: Maxent 3.4.1 and R version 4.1.3, packages ‘raster’, ‘rgdal’, ‘KUENM’, ‘fuzzySim’ and ‘sdm’. - Code availability: 10.6084/m9.figshare.20233758 - Data availability: Data is available in Rodríguez-Ramírez et al. (2021). |
| **DATA** | |
| Biodiversity data | - Taxon names: *Fagus mexicana.* - Taxonomic reference system: We followed the updated list of the International Plant Name Index (https://www.ipni.org/n/103333-2) and Tropicos for Missouri Botanical Garden (https://tropicos.org/name/50161780). - Ecological level: species. - Biodiversity data source: We derived data from literature sources, including our earlier compilation (Ehnis 1981; Williams-Linera et al. 2000, Montiel-Oscura 2011; Rodríguez-Ramírez et al. 2018b, 2021). As an independent source, we conducted fieldwork studies on TMCFs from 2007 to 2020 and we complemented this collected information with satellite imagery observation. - Sampling design: 16 occurrence points of *Fagus mexicana*. - Background data: Random, as no information on the sampling process was available. - Clipping: Mexican tropical montane cloud forest from Sierra Madre Oriental. - Cleaning: We removed from our data: i) duplicate occurrence points; and ii) imprecise point occurrences (e.g. coordinates assigned to municipalities). - Details on potential errors and biases: 10,000 randomly generated background points for MaxEnt. |
| Data partitioning | We conducted 100 replicate analyses for Mexican beech based on a 50% bootstrap of ‌occurrence data. |
| Predictor variables | - Predictor variables: - Past: elevation data and 19 bioclimatic variables from seven palaeoclimatic model projections (e.g. CNRM-CM5, IPSL-CM5A-LR, FGOALS-g2, MIROC-ESM, MPI-ESM-P, MPI-CGCM3 y CCSM4). - Present: elevation data, 19 bioclimatic, aridity index, and annual evapotranspiration variables. - Future: 19 bioclimatic variables for the five models for future climate projections (GFDL-ESM4, IPSL-CM6A-LR, MPI-ESM1-2-HR, MRI-ESM2-0, and UKESM1-0-LL), selected two future climate scenarios (Shared Socioeconomic Pathways; SSP 3-7.0 and 5-8.5). - Data sources: We downloaded elevation data and bioclimatic variables from CHELSA database (<http://chelsa-climate.org/>; Karger et al. 2022), aridity index, and annual evapotranspiration from CGIAR-CSI website (<www.cgiar-csi.org>; Trabucco and Zomer 2019). - Spatial extent: bioclimatic, elevation, aridity index, and annual evapotranspiration variables (Worldwide). - Spatial resolution: environmental variables (30 arc-second or ~1 km spatial resolution). - Temporal extent: past bioclimatic variables (Last Glacial Maximum, ~ 21,000 years), current (1980-2010) and future (2040-2070). - Map projection: WGS 1984 - Dimension reduction: We reduced dimension with the False Discovery Rate calculation (FDR), the multicollinearity degree (*r* <0.7), coefficient of determination of linear regression, tolerance, and the variance inflation factor (VIF), the Bayesian information criterion (BIC), Akaike (AIC), Jackknife analysis, and our knowledge of Mexican beech responses to specific environmental factors. |
| **MODEL** | |
| Multicollinearity | We performed a Pearson’s correlation test to check for multicollinearity among the environmental variables. |
| Model settings | Maxent: default from KUENM package (Cobos et al. 2019) that uses presence-only data and 10,000 randomly generated background points. |
| Model estimates | We used the determination coefficient of linear regression, tolerance, and the variance inflation factor (VIF), the Bayesian information criterion (BIC), Akaike (AIC), and Jackknife analysis. |
| Model selection | We used the average performance evaluation indicators (AUC), Partial ROC (Receiver Operating Characteristic), omission rate, and the optimal complexity parameter (AIC-Akaike Information Criterion). |
| Non-independence correction/analysis | None. |
| Threshold selection | We used a logistic threshold of training presence clipping, which corresponds to the 10% of data with the lowest probability value, commonly used in conservation studies (Abba et al. 2012), and we created a binary map from each modeling output. |
| **ASSESSMENT** | |
| Performance statistics | - Model evaluation (AUC with 95%-CI, partial ROC, omission rate, and optimal complexity parameter-ΔAIC). - Model validation by bootstrapping with 10000 iterations (AUC, calibration plot). We checked for spatial autocorrelation (correlograms). |
| Plausibility check | We checked model plausibility by assessing partial dependence plots. |
| **PREDICTION** | |
| Prediction output | We predicted the potential Mexican beech presence.  We included 10,000 bias files and environmental variables to assess potential habitat suitability analysis. We performed a Gaussian Kernel (Zhang et al. 2018) with QGIS software to avoid the sampling bias effect and identify the highest potential suitability areas, to visualize the areas that are congruent between the predicted models and suitability areas. |
| Uncertainty quantification | N/A |
|  |  |

Appendix S4. Contribution of the bioclimatic variables determining the potential Mexican beech distribution. A= *present* vs. *past* climate; and B= *present* vs. *future* climate.


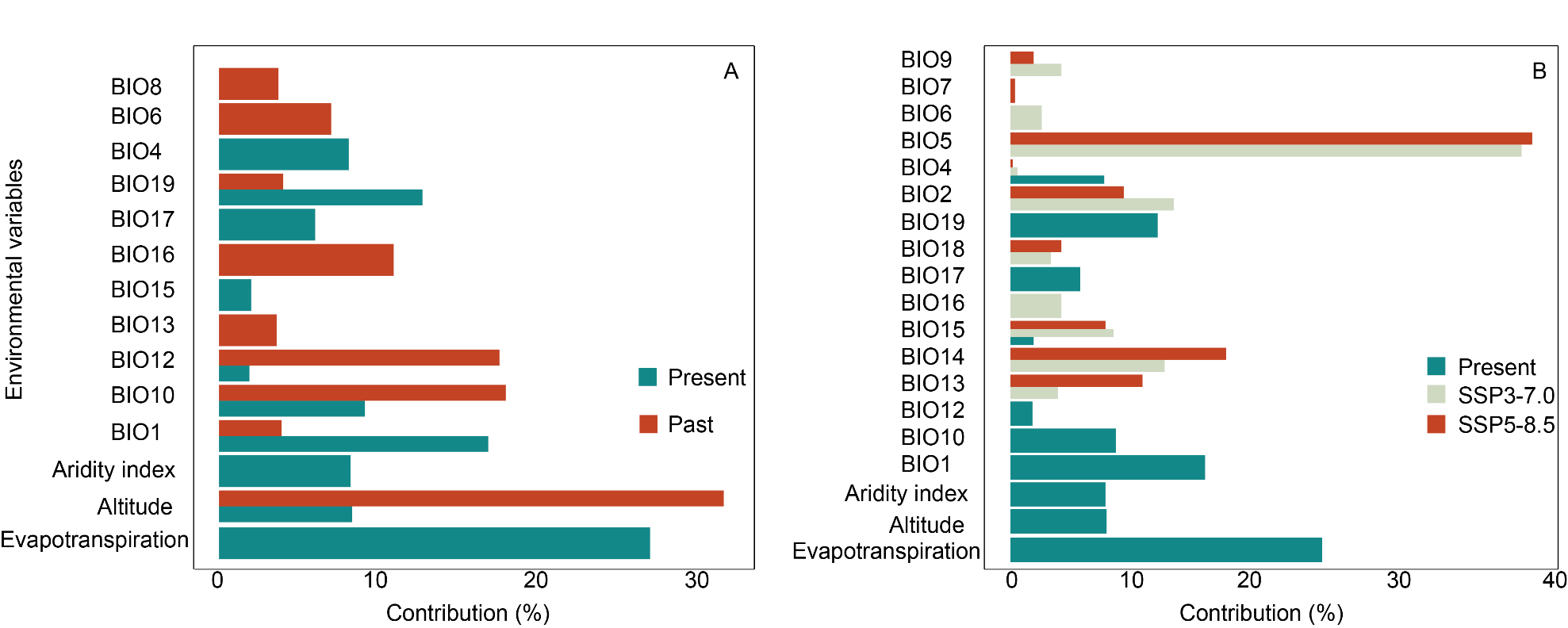


11

Appendix S5. Current and potential distribution of Mexican beech forests, according to environmental variables selected.


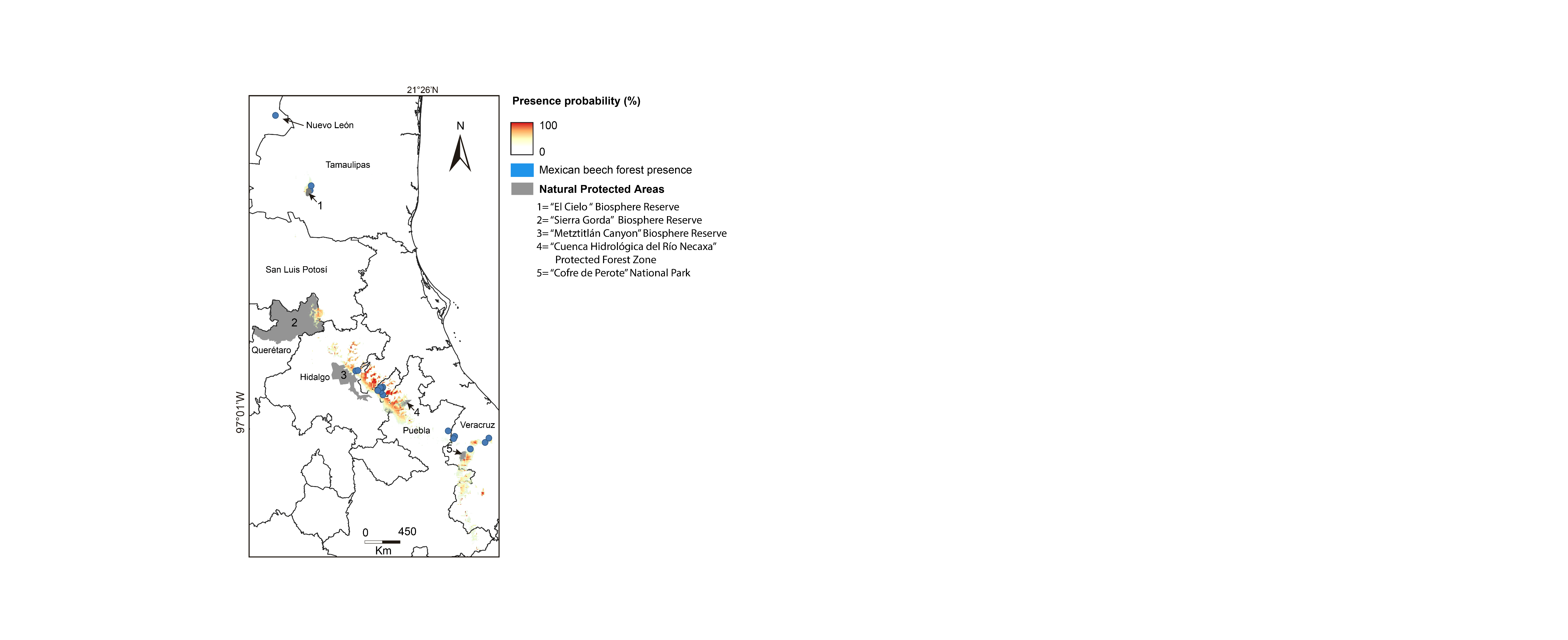


12

Appendix S6. *Future* Mexican beech distribution models according to bioclimatic variables from five general circulation models (ESM4, IPSL-cm6a-lr, MPI-esm-1-2-hr, MRI-esm-2-0 and UKESM1-0-II) under two SSP (Shared Socioeconomic Pathways) of greenhouse gases (SSP3-7.0 and SSP5-8.5). * represents models selected according to statistical indicators (AUC, AICc, Delta AICc and WAICc).


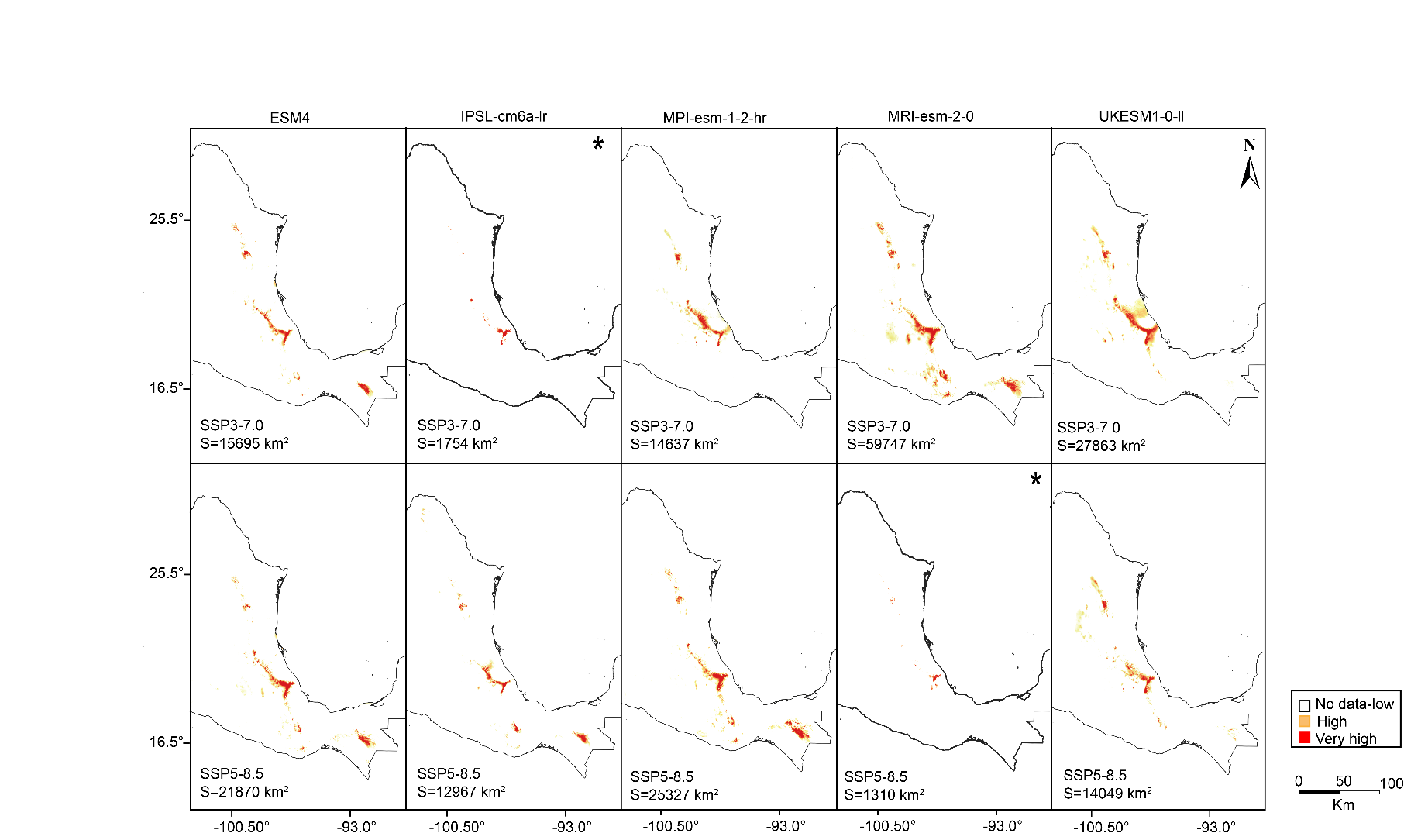


13
